# The laforin/malin E3-ubiquitin ligase complex ubiquitinates pyruvate kinase M1/M2

**DOI:** 10.1186/s12858-015-0053-6

**Published:** 2015-10-23

**Authors:** Rosa Viana, Pablo Lujan, Pascual Sanz

**Affiliations:** Instituto de Biomedicina de Valencia, CSIC, and Centro de Investigación Biomédica en Red de Enfermedades Raras (CIBERER), Jaime Roig 11, 46010 Valencia, Spain

**Keywords:** Laforin, Malin, Ubiquitination, Pyruvate kinase, Nuclear localization

## Abstract

**Background:**

Lafora disease (LD, OMIM 254780) is a fatal neurodegenerative disorder produced mainly by mutations in two genes: *EPM2A*, encoding the dual specificity phosphatase laforin, and *EPM2B*, encoding the E3-ubiquitin ligase malin. Although it is known that laforin and malin may form a functional complex, the underlying molecular mechanisms of this pathology are still far from being understood.

**Methods:**

In order to gain information about the substrates of the laforin/malin complex, we have carried out a yeast substrate-trapping screening, originally designed to identify substrates of protein tyrosine phosphatases.

**Results:**

Our results identify the two muscular isoforms of pyruvate kinase (PKM1 and PKM2) as novel interaction partners of laforin.

**Conclusions:**

We present evidence indicating that the laforin/malin complex is able to interact with and ubiquitinate both PKM1 and PKM2. This post-translational modification, although it does not affect the catalytic activity of PKM1, it impairs the nuclear localization of PKM2.

## Background

Lafora disease (LD, OMIM 254780) is a rare fatal type of progressive myoclonus epilepsy that initially manifests during adolescence. Recessive mutations in two main loci, *EPM2A* or *EPM2B,* are known to produce LD ([1–3]). The *EPM2A* gene encodes the dual-specificity phosphatase laforin and the *EPM2B* gene encodes the E3-ubiquitin ligase malin. Although it is known that both proteins form a functional complex ([4–6]), the underlying molecular mechanisms of this pathology are still far from being understood. For this reason the search for substrates of the laforin/malin complex has been a good strategy to understand the molecular basis of this pathology. Different techniques have been used with this aim, from regular yeast two-hybrid screening, using either laforin or malin as bait, to co-immunoprecipitation/pull down analyses and functional interactions. All these approaches have identified several putative substrates of the laforin/malin complex that have enriched our knowledge on the different cellular pathways where laforin and malin may participate (see [7] for a review).

**Fig. 1:**
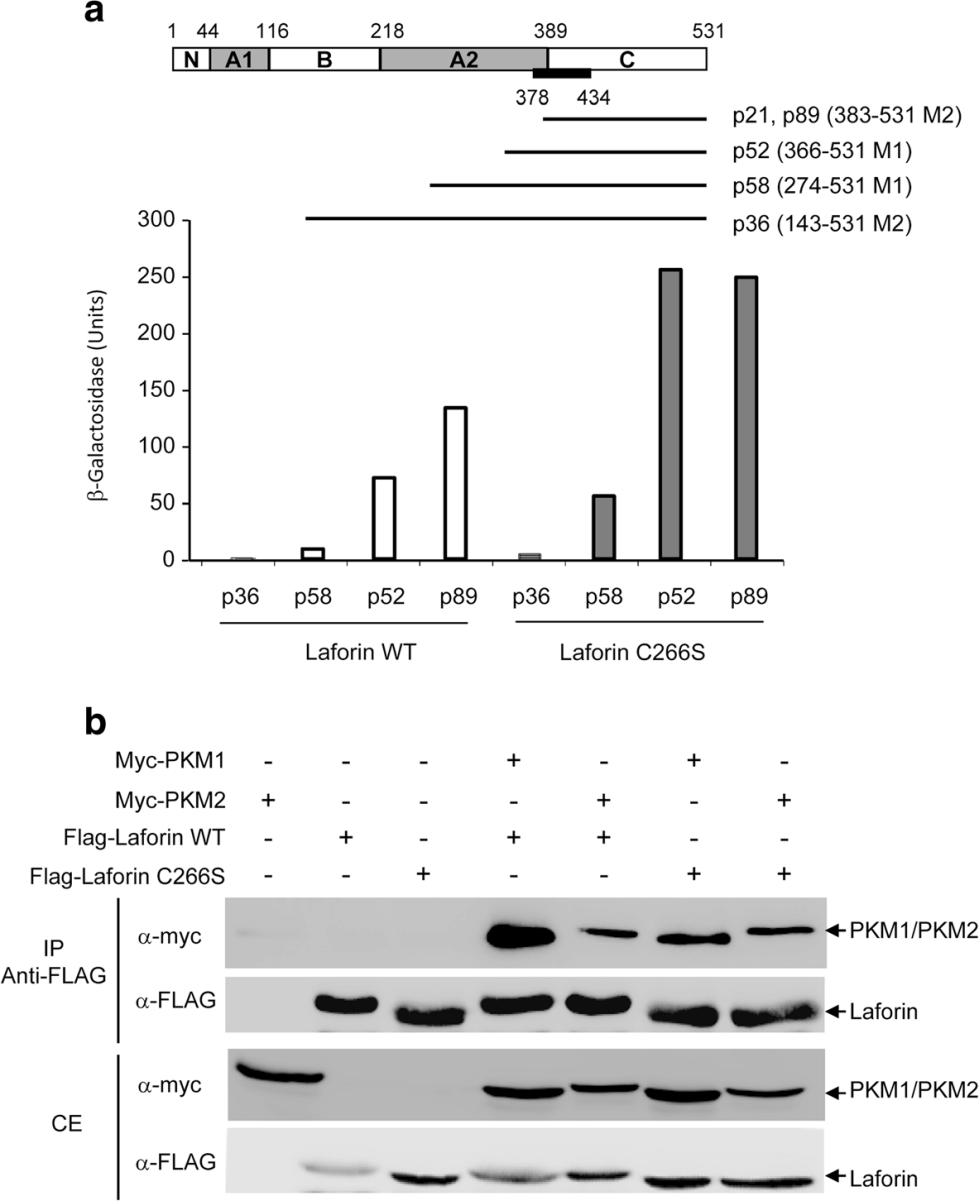
PKM1 and PKM2 interact with laforin. **a** Yeast substrate-trapping system with LexA-laforin C266S was used to identify putative partners from a human brain library. Five clones corresponding to different lengths of PKM1 and PKM2 were selected (in brackets the amino acids included in the clones corresponding either to PKM1 of PKM2). A diagram of the different domains in PKMs is presented (black bar indicates the region that is different between PKM1 and PKM2). The strength of the interaction of these clones with either LexA-laforin C266S or LexA-laforin wild type was measured (β-galactosidase activity). **b** Co-immunoprecipitation analysis of PKM1 and PKM2 with laforin. HEK293 cells were transfected with the indicated plasmids. Crude extracts and co-immunoprecipitation analyses with anti-FLAG antibody was performed as indicated in Methods. Proteins in the immunoprecipitates and in the crude extracts (CE, 25 μg) were analyzed by western blotting using the indicated antibodies

In order to identify novel phospho-protein substrates of laforin, we have used the yeast substrate-trapping system described by Fukada and Noda [8], designed to identify substrates of protein tyrosine phosphatases. This technique is based on the use of phosphatase mutants which harbor a substitution in invariant amino acid residues in the catalytic domain, making them catalytically inactive, but retaining the capability to recognize the phosphorylated substrate. In addition, the yeast model co-expresses a wide spectrum tyrosine kinase (i.e., v-src), which will phosphorylate putative target substrates. In this way, the possibilities to identify putative phospho-tyrosine containing substrates of a particular phosphatase are enhanced. Here, we have identified the two muscular isoforms of pyruvate kinase (PKM1 and PKM2) as novel interaction partners of laforin. Pyruvate kinase regulates the rate-limiting final step of glycolysis, which catalyzes the transfer of a high-energy phosphate group from phosphoenolpyruvate (PEP) to adenine diphosphate (ADP), yielding one molecule of pyruvate and one molecule of adenosine triphosphate (ATP) (see [9] for review). Four isoforms of pyruvate kinase have been described in mammals: PKR, present mainly in erythrocytes; PKL, present in liver, kidney, small intestine and pancreas; PKM1, present in muscle, heart and brain; and PKM2, widely distributed and also present in fetal tissues and cancer cells ([9, 10]). PKM1 and PKM2 result from an alternative splicing of two mutually exclusive exons of the *PKM* gene (exon 9 is present in PKM1 whereas exon 10 is present in PKM2). This difference in exon usage results in proteins that differ only in 22 amino acids distributed along a small region of 56 amino acids [from residue 378 to 434 [11], (Fig. 1a, black box)]. These changes confer distinct kinetic and regulatory properties to PKM1 and PKM2: although both PKM1 and PKM2 are proteins of 531 aa, with a similar molecular weight (58 kDa) and similar structural domains ([9, 10]) (Fig. 1a), only PKM1 is fully active whereas PKM2 may form inactive dimeric complexes ([9, 10]).

In this work we present evidence of the interaction between laforin and both PKM1 and PKM2. As a result of this interaction, we show that the laforin/malin complex ubiquitinates PKM1 and PKM2 and that this post-translational modification, although it does not affect the catalytic activity of PKM1, it impairs the nuclear localization of PKM2.

## Results

### The muscular isoform of Pyruvate Kinase (PKM) interacts with laforin

As indicated at the Background section, in this work we have used the yeast substrate-trapping system [8], designed to identify substrates of protein tyrosine phosphatases, to screen for possible phospho-protein substrates of laforin. Following the conditions required for this type of screening, we initially constructed a catalytically inactive laforin mutant, Laf-C266S, which affects the key Cys residue in the catalytic site [7]. This mutant retained the capability to recognize laforin binding partners (i.e. malin, R5/PTG) (not shown). A yeast strain co-expressing LexA-Laforin C266S and the protein tyrosine kinase v-src was transformed with a human brain cDNA library, and after screening more than 300,000 independent transformants, we recovered 97 putative positive clones. Unfortunately, 58 of them were false positives since when we purified the corresponding plasmids they were not able to reproduce the two-hybrid interaction. Among the other 39 positive clones, we identified already known laforin binding partners, such as laforin itself and R5/PTG, what validated the technique (Table 1). The rest of the clones interacted with laforin with different strength (in Table 1 we describe only the 17 clones that presented the strongest interaction). Among them, we focused our attention to those that coded for the two muscular isoforms of pyruvate kinase (PKM1 and PKM2), as we recovered five PKM-related clones which encoded for different fragments of both PKM1 and PKM2 (Fig. 1a). In these clones the interaction with laforin was much stronger when only the C-terminal part of PKMs was present in the clones [clones p21 and p89 (aa 383–531 of PKM2) and p52 (aa 366–531 of PKM1)], and decreased when larger parts of the protein were present in the clones [clone p58 (aa 274–531 of PKM1), clone p36 (aa 143–531 of PKM2)] (Fig. 1a). We repeated the two-hybrid interaction of these clones using LexA-Laforin WT as bait and found that the interaction was maintained although at lower intensity (Fig. 1a). We also noticed that all these interactions were maintained under growth conditions that prevented the expression of v-src, indicating that, contrary to the experimental design, they were not dependent on the phosphorylation of the substrate by this protein kinase.

**Table 1:** Positive clones identified in substrate-trap screening of a human brain cDNA library using LexA-laforin C266S as bait. Yeast THY-AP4 cells transformed with plasmid pBridge-laforin C266S were co-transformed with a human brain cDNA library in pACT2

No. of positive clones	Encoded protein	Uniprot number	Intensity of interaction
5	Pyruvate kinase PKM1/PKM2	Q504U3	From ++ to +++
3	E3 ubiquitin ligase TOPORS	Q9NS56	++
3	Cytochrome C oxidase subunit 5A	P20674	++
1	Laforin (EPM2A)	O95278	+++
1	PP1 regulatory subunit R5/PTG (PPP1R3C)	Q9UQK1	++
1	G/T mismatch-specific thymine DNA glycosylase	Q13569	+++
1	26S proteasome regulatory subunit 6A	P17980	++
1	HMG domain-containing protein 4	Q9UGU5	++
1	Translation initiation factor eIF-2B subunit alpha	Q14232	++

Positive clones were confirmed, subjected to BLAST analysis and protein interaction estimated by β-galactosidase filter lift assays (+++, when the blue color appeared in less than 5 min; ++, when the blue color appeared between 5 and 20 min)

In order to confirm the interaction between laforin and the PKM1 and PKM2 isoforms, we carried out a co-immunoprecipitation analysis in mammalian cells. First, we obtained by PCR the full length cDNAs of PKM1 and PKM2 from the human brain cDNA library and subcloned them into plasmid pCMV-myc, to allow their expression in mammalian HEK293 cells. We observed that the expression of the PKM1 isoform yielded a protein with a slightly faster mobility in SDS-PAGE, probably due to differences in protein sequence in comparison to PKM2 (Fig. 1b. third panel). We co-expressed in HEK293 cells the PKM1 and PKM2 isoforms and laforin (tagged with a FLAG epitope) either wild type or C266S, immunoprecipitated the crude extracts with anti-FLAG antibody and analyzed the immunoprecipitates by western blotting using anti-FLAG and anti-myc antibodies. As shown in Fig. 1b (top panel) both laforin-WT and laforin-C266S were able to co-immunoprecipitate both myc-PKM1 and myc-PKM2 isoforms. Taken together, these results indicate that laforin interacts with both PKM1 and PKM2, and that the phosphatase activity of laforin is dispensable for the interaction with the PKMs isoforms. We tried to repeat the co-immunoprecipitation experiment with endogenous levels of proteins but unfortunately we did not find any reliable commercial antibody which recognized endogenous levels of laforin.

### The laforin/malin complex polyubiquitinates PKM isoforms, attaching K63-linked ubiquitins

**Fig. 2:**
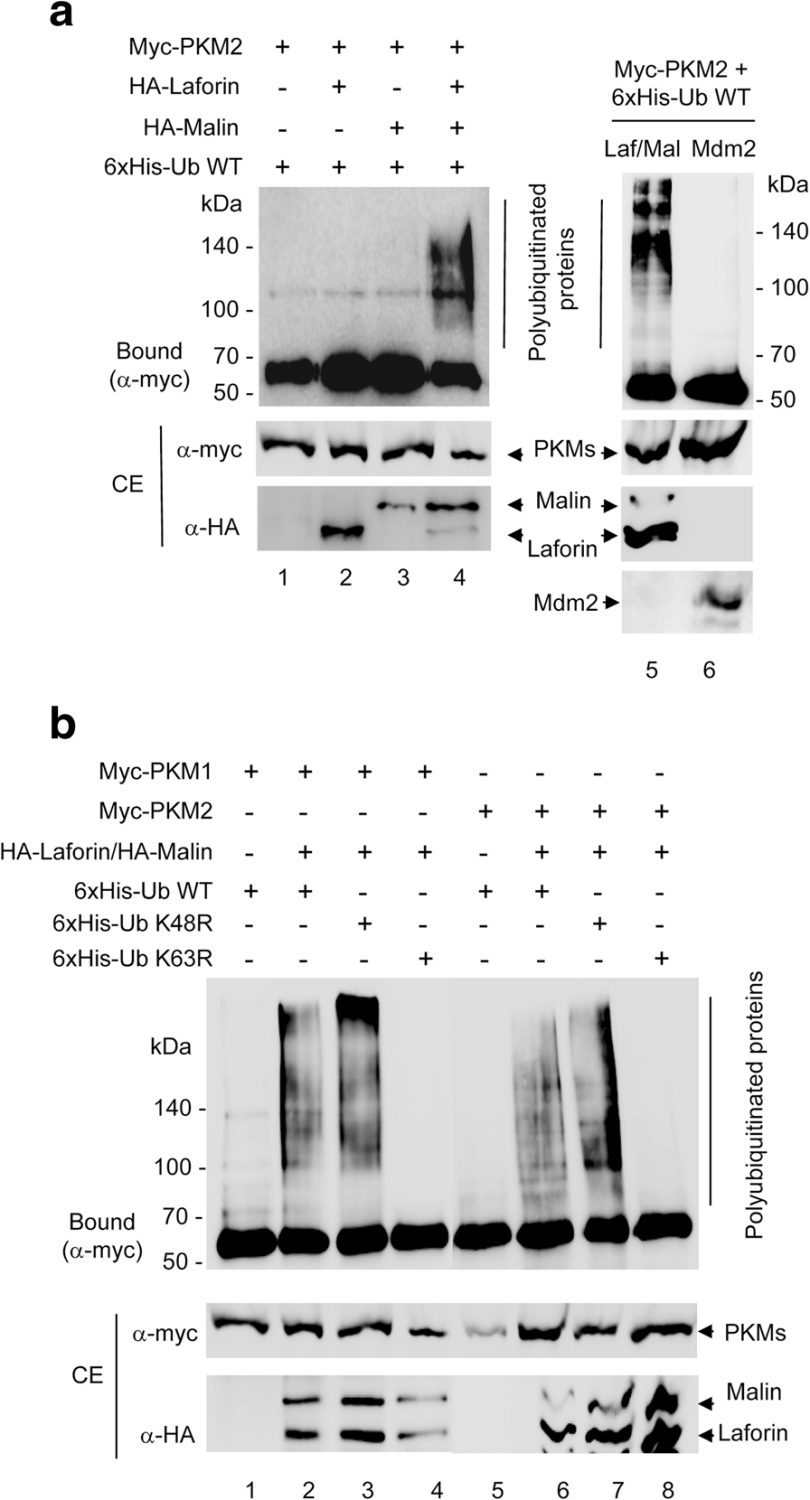
The laforin/malin complex polyubiquitinates PKM isoforms attaching K63-linked ubiquitins. **a** and **b** HEK293 cells expressing the indicated plasmids were analyzed for in vivo ubiquitination as indicated in Methods. Proteins bound to the metal affinity column (Bound) and those present in the crude extracts (CE, 25 μg) were analyzed by western blotting using the indicated antibodies. The position of polyubiquitinated proteins is indicated. The fuzzy bands in the CE are probably due to the presence of 6 M guanidinium hydrochoride in the extracts

It is known that laforin forms a functional complex with the E3-ubiquitin ligase malin, in which laforin recognizes the substrates that are eventually ubiquitinated by malin. In order to check if the interaction between PKM1 and PKM2 and laforin resulted in the ubiquitination of the PKM isoforms, we co-expressed in HEK293 cells myc-PKM1 or myc-PKM2, laforin, malin and a 6xHis-tagged ubiquitin. The 6xHis tag present in the ubiquitin allows the recovery of polyubiquitinated forms by metal affinity chromatography in the presence of guanidinium hydrochloride, an inhibitor of the action of deubiquitinases [12]. As shown in Fig. 2a, the laforin/malin complex was able to promote the polyubiquitination of PKM2 (lane 4). This modification was dependent on the presence of the laforin/malin complex, since it was drastically reduced in its absence (lane 1) or when only laforin or malin were expressed in the cells (lanes 2 and 3 respectively). Similar results were obtained when PKM1 was assayed in the same way (not shown). This ubiquitination was specific of the laforin/malin complex since we did not observe this modification when we co-expressed Mdm2, an unrelated E3-ubiquitin ligase [lane 6 in Fig. 2a for PKM2; similar results were obtained for PKM1 (not shown)].

In order to analyze the topology of the laforin/malin-induced polyubiquitinated chains in PKMs, we co-expressed in HEK293 cells mutated forms of ubiquitin that prevented the formation of ubiquitin chains in either K48 (K48R mutant) or K63 (K63R mutant) (K48-linked polyubiquitin chains signal proteins to be degraded by the proteasome, whereas K63-linked polyubiquitin chains play a role in cell signaling and other physiological processes). As shown in Fig. 2b, in the presence of K48R-ubiquitin, a similar pattern of polyubiquitination of PKM1 and PKM2 by the laforin/malin complex was obtained (lanes 3 and 7, respectively), whereas in the presence of K63R-ubiquitin, no polyubiquitinated forms were obtained (lanes 4 and 8, respectively). These results indicate that the laforin/malin complex promotes the polyubiquitination of PKM1 and PKM2 through the attachment of K63-linked ubiquitin moieties. This topology was similar to the one found in other substrates of the laforin/malin complex, such as R5/PTG and AMPK subunits [13], and suggests that the modified PKMs may be involved in cellular functions not related to proteasomal degradation.

### Consequences of the action of the laforin/malin complex on the biochemical properties of PKM1 and PKM2

**Fig. 3:**
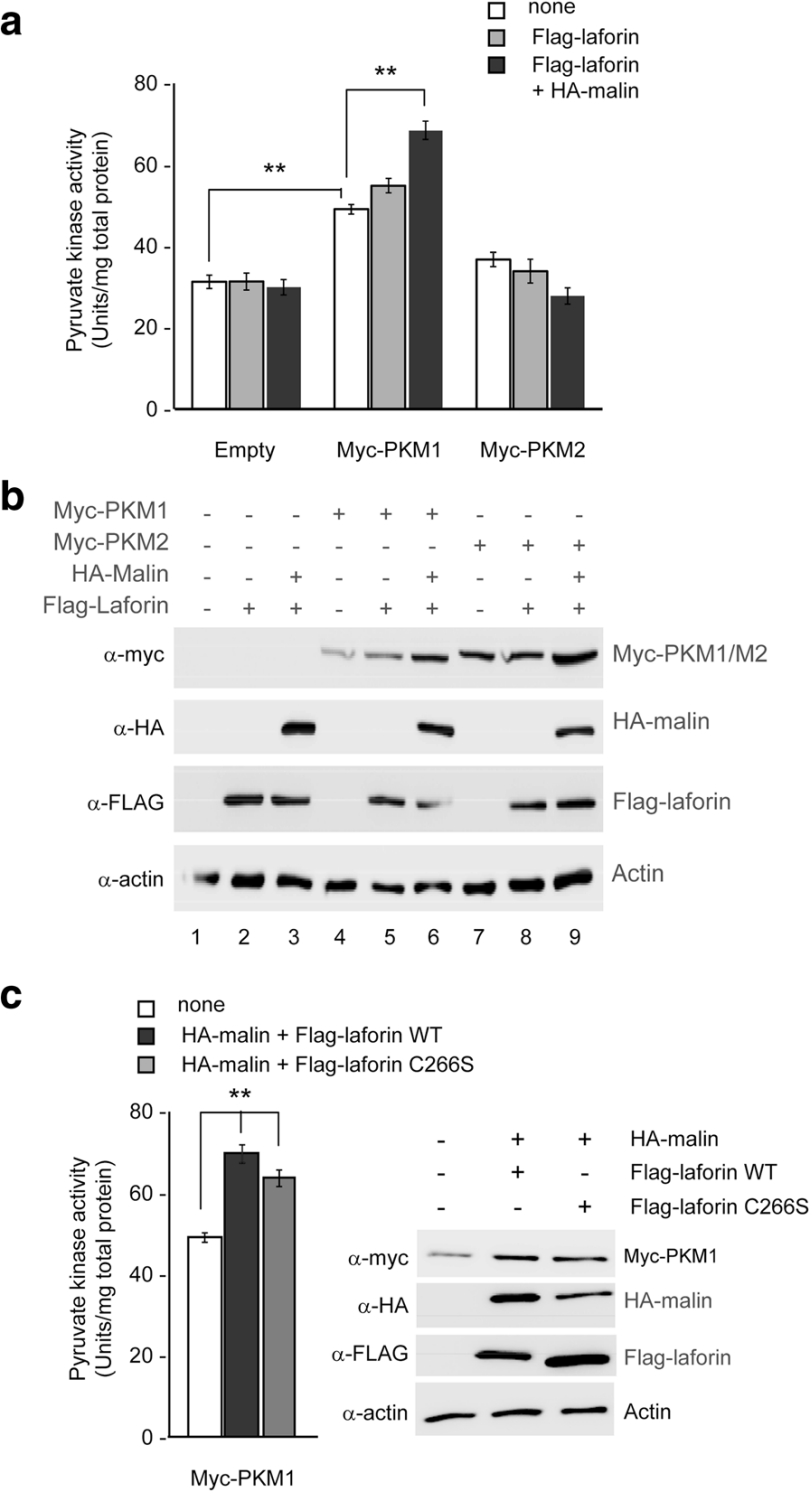
Effect of the laforin/malin complex on the enzymatic activity of the PKM isoforms. **a** Crude extracts from HEK293 cells expressing the indicated plasmids were used to determine the pyruvate kinase enzymatic activity as indicated in Methods. The results were plotted as Units/mg total protein in the extracts (data are the mean of at least three independent experiments; bars indicate standard deviation; **p < 0.01). **b** Crude extracts from panel **a** were also analyzed by SDS-PAGE and immunoblotting using the indicated antibodies. **c** HEK293 cells expressing myc-PKM1 were transfected with the indicated plasmid. Crude extracts were obtained and the pyruvate kinase enzymatic activity measured as in panel **a**) (data are the mean of at least three independent experiments; bars indicate standard deviation; **p < 0.01). Crude extracts were also analyzed by SDS-PAGE and immunoblotting using the indicated antibodies (*right panel*)

We analyzed next the effect of the laforin/malin complex on the enzymatic activity of PKMs. With this aim, the pyruvate kinase activity present in HEK293 cells expressing PKM1 or PKM2 and co-expressing or not the laforin/malin complex was measured by standard methods. As expected, the overexpression of PKM1 increased the pyruvate kinase activity over endogenous levels (expressed as Units/mg total protein in the extract) (Fig. 3a, white bars). The activity in the extract was increased further if the cells co-expressed the laforin/malin complex (Fig. 3a, black bars) but the co-expression of laforin alone (Fig. 3a, grey bars) did not have this effect. On the contrary, the overexpression of PKM2 did not enhance the pyruvate kinase activity over endogenous levels (Fig. 3a) (what was in agreement with the reported reduced activity of this isoform [10]), and the laforin/malin complex had no effect on its activity (Fig. 3a). Although at first glance these results could suggest that the laforin/malin complex increased the intrinsic enzymatic activity of PKM1, we think that this is a misleading conclusion, since the steady-state levels of PKM1 were higher in cells expressing the laforin/malin complex (Fig. 3b, compare lane 4 with lane 6). So, we think that the increase in the total pyruvate kinase activity detected in cells co-expressing PKM1 and the laforin/malin complex is just a reflection of more amount of the PKM1 protein being present in the extracts. We also observed that the laforin/malin complex did not modify the total endogenous enzymatic activity of PKM isoforms present in Hek293 cells (transformed with an empty vector; Fig. 3a, empty).

We repeated the assay with cells expressing the catalytically inactive laforin C266S form. As shown in Fig. 3c, the laforin C266S/malin complex was also able to increase the total enzymatic activity and the steady-state levels of PKM1 in the cells, indicating that this effect was not dependent on the phosphatase activity of laforin.

### Altered subcellular location of PKM2 due to the laforin/malin complex

**Fig. 4:**
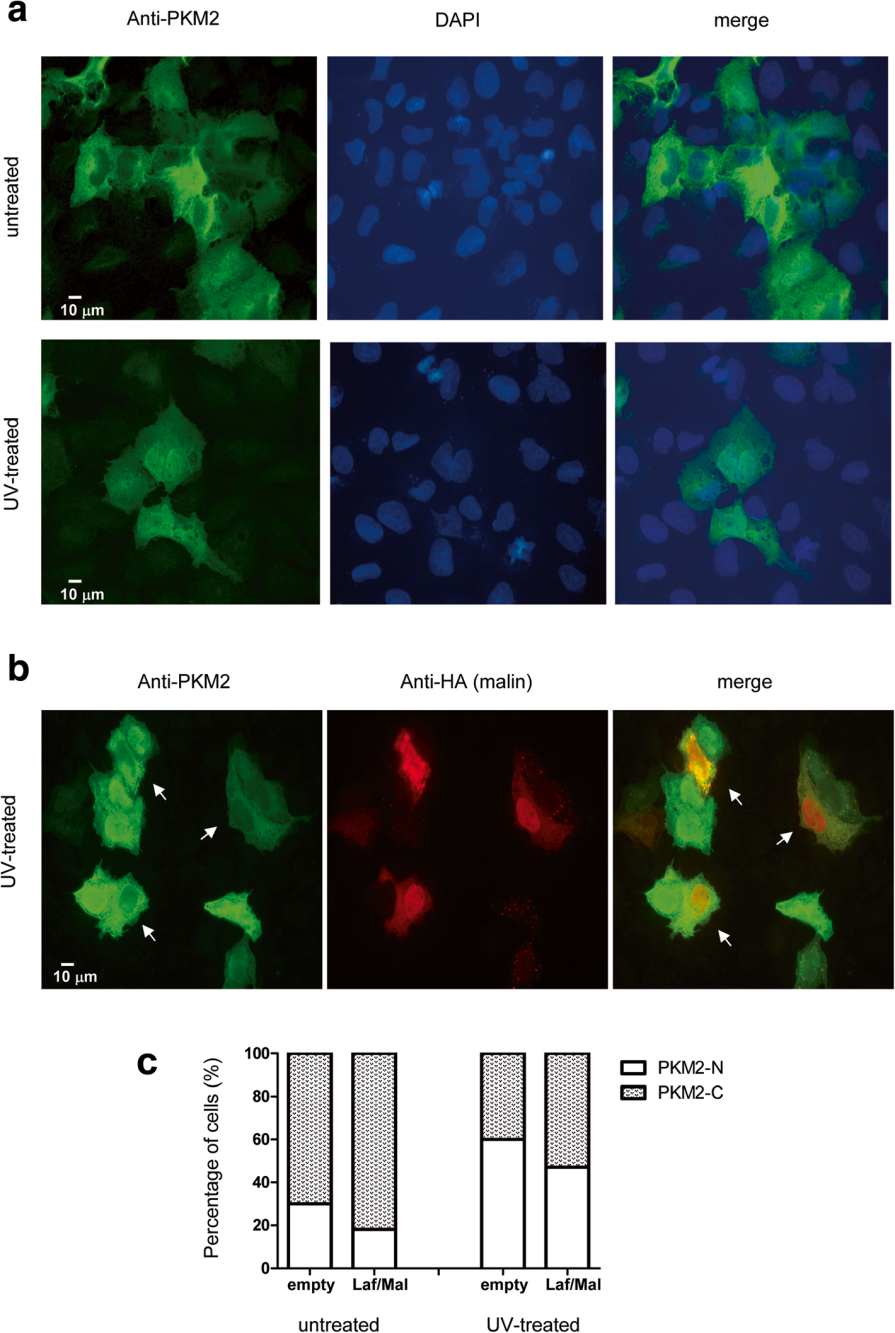
The laforin/malin complex alters the subcellular localization of PKM2. **a** U2OS cells were transfected with plasmid pCMV-myc-PKM2. Cells were then treated or not with UV light (120 mJ/cm^2^; 120 s) to induce the accumulation of PKM2 into the nucleus. The localization of PKM2 was determined by immunofluorescence using anti-PKM2 antibodies (see Methods). DAPI staining was also performed to define the position of the nuclei. A merge image is presented. Scale bars are indicated. **b** U2OS cells expressing plasmid pCMV-myc-PKM2 were co-transfected with plasmids pFLAG-laforin and pCMV-HA-malin. Then, cells were treated of not with UV light as above. Panel shows the result in UV-treated cells. The localization of malin, a nuclear protein, and PKM2 was determined by immunofluorescence using anti-HA and anti-PKM2 antibodies, respectively. **c** The percentage of cells having a nuclear localization of PKM2 was determined in 100 cells from all the conditions. PKM-N: cells that present PKM2 inside the nucleus; PKM-C: cells that present PKM2 outside of the nucleus

It has been described that both PKM1 and PKM2 are cytosolic proteins, what is consistent with their role in the glycolytic pathway ([14, 15]). However, PKM2 but not PKM1 is also able to translocate to the nucleus, where it acts as a protein kinase and regulates gene expression in different ways ([16–18]). In order to check whether the ubiquitination of PKM2 by the laforin/malin complex could affect its translocation to the nucleus, we transfected human osteosarcoma U2OS cells [which are larger than HEK293 cells, so the different subcellular compartments (especially nucleus) are better observed] with myc-PKM2 in the presence or absence of FLAG-laforin and HA-malin. Transfected cells were treated or not with UV light (120 mJ/cm^2^; 120 s) to induce the accumulation of PKM2 into the nucleus [19]. Cells were then analyzed by immunofluorescence using anti-PKM2 and anti-HA (to detect the localization of malin, a protein enriched in the nucleus) antibodies. In agreement with previous reports [19], treatment with UV light of the cells containing myc-PKM2 and transformed with an empty HA-vector promoted the nuclear accumulation of PKM2 (Fig. 4a); 60 % of the cells presented a nuclear accumulation of PKM2 after UV-treatment in contrast to 30 % in the untreated sample (Fig. 4c). However, the expression of the laforin/malin complex produced a reduction in the number of cells containing PKM2 at the nucleus in both UV treated and untreated cells [Fig. 4b; compare cells expressing malin (white arrows) with other cells that do not express malin]: in untreated cells, only 18 % of cells expressing the laforin/malin complex displayed a nuclear accumulation of PKM2, in contrast to 30 % in cells containing an empty plasmid, and in UV-treated cells, only 47 % of cells expressing the laforin/malin complex had a nuclear PKM2, in contrast to 60 % in cells containing an empty plasmid (Fig. 4c).

Taken all these results together, we suggest that the laforin/malin dependent ubiquitination of PKM2 partially impairs its nuclear localization.

## Discussion

In this work we describe additional substrates of the laforin/malin complex, namely the muscular isoforms of pyruvate kinase (PKM1 and PKM2). We present evidence of the interaction between laforin and both PKM1 and PKM2, and as a result of this interaction, we show that the laforin/malin complex ubiquitinates both PKM1 and PKM2 by attaching K63-linked polyubiquitin chains. These results are in agreement with recent data from the GGBase indicating that PKM2 was ubiquitinated at different sites (https://gygi.med.harvard.edu/ggbase/) and indicate for the first time that in the ubiquitination reaction participates, at least, the laforin/malin E3-ubiquitin ligase complex. Therefore, ubiquitination of PKMs should be added to the list of post-translational modifications that suffer these proteins [14]. The laforin/malin dependent ubiquitination of PKM1 and PKM2 did not modify their corresponding catalytic activities. However, we noticed that this post-translational modification increased the steady-state levels of the proteins, probably because the modified forms were more resistant to their proteolytic turnover. In this way, the effect of the laforin/malin dependent modification of PKM1 and PKM2 resembles that of AMPKβ subunits (other substrates of the laforin/malin complex) since also higher steady-state levels of these subunits were observed upon the action of the laforin/malin complex [13]. It is worth to point out that stabilization of PKM1 and PKM2 levels also occurs when a phosphatase inactive form of laforin (laforin C266S) is expressed, indicating that the phosphatase activity of laforin may not be required for inducing the malin-mediated polyubiquitination of PKM1 and PKM2, as already demonstrated in other laforin/malin substrates, such as R5/PTG or R6, two regulatory subunits of protein phosphatase type1 ([6, 20]).

We next studied the effect of the laforin/malin dependent polyubiquitination on the subcellular distribution of PKMs. It has been described that PKM2 but not PKM1 is able to translocate to the nucleus, where it displays specific functions ([14, 15]). This is probably due to the presence of a nuclear localization signal (NLS) in the specific region (aa 378 to 434) of PKM2, not present in PKM1. This NLS allows PKM2 to interact with members of the importin family, who mediate in the translocation of PKM2 to the nucleus [16]. Recently, it was reported that PKM2 interacted with the E3-SUMO ligase PIAS3 and this resulted in the sumoylation of PKM2 and in an enhancement of its nuclear localization [21]. On the contrary, our results suggest that the laforin/malin dependent ubiquitination of PKM2 interferes with the localization of PKM2 to the nucleus. Perhaps ubiquitination of PKM2 affects its recognition by the importin machinery, resulting in an impairment of nuclear translocation. Alternatively, ubiquitination of PKM2 could interfere with its sumoylation since both processes affect similar Lys residues and, in some cases, the introduction of one modification (i.e. ubiquitination) prevents the alternative post-translational alteration by the other (sumoylation) ([22, 23]). This negative relationship between ubiquitination and sumoylation might affect nuclear translocation of PKM2, as it has already been documented in other cases [24].

## Conclusions

We present evidence indicating that the laforin/malin complex is able to interact with and ubiquitinate both muscular isoforms of pyruvate kinase (PKM1 and PKM2). This post-translational modification, although it does not affect the catalytic activity of PKM1, it impairs the nuclear localization of PKM2.

## Methods

### Microorganisms, culture conditions and genetic methods

*Escherichia coli* DH5α strain was used as the host strain for plasmid constructions and protein production. It was grown in LB (1 % peptone, 0.5 % yeast extract, 1 % NaCl, pH 7.5) medium supplemented with 50 mg/l ampicillin. Yeast strain used in this work was THY-AP4 (*MAT*a, *ura3, leu2, lexA::lacZ::trp1, lexA::HIS3, lexA::ADE2*). Yeast transformation was carried out using the lithium acetate protocol [25]. Yeast cultures were grown in synthetic complete (SC) medium lacking the corresponding supplements to maintain selection for plasmids [26].

### Plasmids

pCMV-HA-laforin, pFLAG-laforin, pFLAG-laforin C266S and pcDNA3-HA-malin plasmids were described previously [6]. Plasmids pBridge-laforin and pBridge-laforin C266S were obtained by subcloningBamHI/SalI fragments containing either laforin WT or C266S in pBridgeLexA/v-src [8]. Plasmids pCMV-myc-PKM1 and pCMV-myc-PKM2 were obtained by subcloning an EcoRI/XhoI fragment containing the corresponding ORF in pCMV-myc vector. Other plasmids used in this study were pCMV-6xHisUbiq (from Dr. M. Rodriguez, Proteomics Unit, CIC-BioGUNE, Vizcaya, Spain); pCMV-6xHisUbiq-K48R and pCMV-6xHisUbiq-K63R (from Dr. Ch. Blattner, Institute of Toxicology and Genetics, Karlsruhe Institute of Technology, Karlsruhe, Germany); pCMV-Mdm2 (from Dr. M. Gentry, University of Kentucky, Lexington, KY; [27]).

### Two-hybrid screening and analysis

A modified form of yeast two-hybrid screening named yeast substrate-trapping system [8] was used to identify proteins that interacted with LexA-laforin C266S (plasmid pBridge-laforin C266S). Plasmid pBridge-laforin C266S also contains the ORF corresponding to the mammalian protein kinase v-src under the control of the MET25 promoter. *Saccharomyces cerevisiae* THY-AP4 strain containing pBridge-laforin C266S plasmid was transformed with a commercial human brain cDNA library in pACT2 vector (Clontech, Madrid, Spain). Transformants were selected in SC + 2 % glucose plates lacking tryptophan, leucine, methionine and histidine and were subsequently screened for β-galactosidase activity using a filter lift assay [28]. pACT2-plasmids were recovered from positive clones and used to transform again *S. cerevisiae* THY-AP4 strain containing pBridge-laforin C266S or laforin WT, to confirm the interaction. The strength of the interaction was determined by measuring β-galactosidase activity in permeabilized yeast cells and expressed in Miller units as described by Ludin and collaborators [29].

### Cell culture, transfection and preparation of crude extracts

Human embryonic kidney (HEK293) and human osteosarcoma (U2OS) cells were grown in Dulbecco’s modified Eagle’s medium (Lonza, Barcelona, Spain), supplemented with 100 units/ml penicillin, 100 μg/ml streptomycin, 2 mM glutamine and 10 % of inactivated fetal bovine serum (Lonza, Barcelona, Spain) in a humidified atmosphere at 37 °C with 5 % CO_2_. When indicated, cells were transfected with 1 μg of each plasmid using X-treme GENE HP transfection reagent (Roche Diagnostics, Barcelona, Spain) according to the manufacturer’s instructions. Cell extracts were prepared using lysis buffer A [25 mMTrisHCl at pH 7.4, 15 mM EDTA at pH 8, 50 mMNaF, 0.6 M sucrose, 15 mM Na_4_P_2_O_7_, 1 % nonidet P40, 10 mMNaCl, 1 mM PMSF, and a complete protease inhibitor mixture (Roche Diagnostics, Barcelona, Spain)]. Cells were lysed by repeated passage through 24G × 5/8″ needle and whole lysates were centrifuged at 10,000 × g for 15 min. The supernatants were collected and 25 μg of total protein subjected to SDS-PAGE, transferred into PVDF membrane and revealed with the appropriated antibodies.

### Co-immunoprecipitation and western blotting

Mammalian HEK293 cells were transfected with the corresponding plasmids. Twenty-four hours after transfection, cells were harvested with lysis buffer B [50 mMTrisHCl pH 7.5, 10 mMNaCl, 2 mM EDTA, 15 % glycerol, 1 % nonidet P40, complete protease inhibitor cocktail (Roche Diagnostics, Barcelona, Spain) and 1 mM PMSF]. Cells were lysed by repeated passage through 24G×5/8″ needle, whole lysates were centrifuged at 10,000 × g for 15 min and the soluble fraction was collected for immunoprecipitation. Co-immunoprecipitation experiments were carried out with 500 μg of soluble protein extracts, in a final volume of 500 μl of lysis buffer, adding 1 μl of anti-FLAG monoclonal antibodies (Sigma, Madrid, Spain) as in [30]. For western blot analysis, proteins (25 μg) were denatured using sample buffer (125 mMTrisHCl pH 6.8, 20 % Glicerol, 0.01 % bromophenol blue, 4 % SDS) and heating to95 °C for 5 min. The samples were subjected to SDS-PAGE and transferred onto PVDF membranes (Millipore, Madrid, Spain). Membranes were blocked with 5 % milk in TBS-tween for 1 h and incubated with the following specific antibodies: anti-myc, anti-FLAG, anti-HA and anti-actin from Sigma (Madrid, Spain); anti-Mdm2 from Santa Cruz Biotechnology (Madrid, Spain). Thereafter, blots were washed with TBS-tween and further incubated for 1 h with the corresponding secondary antibody conjugated with horseradish peroxidase. Finally, membranes were washed (3× 15 min) with TBS-tween and analyzed by chemiluminiscence (ECL Western Blotting Detection Reagents, GE Healthcare, UK) using an image reader LAS-4000 (GE Healthcare, UK).

### Analysis of ubiquitination

For ubiquitination assays, HEK293 cells were co-transfected with pCMV-myc-PKM1 or pCMV-myc-PKM2, 6xHis-tagged ubiquitin (WT, K48R or K63R) plasmids and, when indicated, with pcDNA3-HA-malin and pCMV-HA-laforin, or pCMV-Mdm2 plasmids, using X-treme GENE transfection reagent, according to the manufacturer’s instructions (Roche Diagnostics, Barcelona, Spain). After 18 h of transfection, cells were lysed in guanidinium hydrochloride to inhibit the action of deubiquitinases and ubiquitinated proteins purified by metal affinity chromatography [12]. Bound proteins and clarified extracts were analyzed by immunoblotting with the appropriated antibodies.

### Determination of pyruvate kinase enzymatic activity

HEK293 cells transfected with the indicated plasmids were resuspended in lysis buffer C [50 mMTrisHClpH 7.5, 1 mM EDTA, 150 mMNaCl, 1%NP-40, 1 mM DTT, 10 μM fructose 1,6 bisphosphateand complete protease inhibitor mixture (Roche Diagnostics, Barcelona, Spain)]. Cells were lysed by repeated passage through 24G × 5/8″ needle and whole lysates were centrifuged at 10,000 × g for 15 min. Clarified extracts were used to determine the pyruvate kinase activity by standard methods (reaction buffer: 40 mM K_2_HPO_4_ pH 7.6, 0.58 mM phosphoenolpyruvate, 0.11 mM NADH, 6.8 mM MgSO_4_, 1.5 mM ADP, 10 units lactate dehydrogenase, 10 μM fructose 1,6 bisphosphate). The disappearance of NADH absorbance was measured spectrophotometrically at 340 nm. One unit of enzymatic activity is defined as the amount of enzyme that is able to oxidize 1 μmol of NADH per 1 min at 25 °C. Enzymatic activity was normalized by the total amount of protein in the sample.

### Immunofluorescence analysis

Human osteosarcoma U2OS cells transfected with the indicated plasmids were grown on 12-well plates containing coverslips. Cells were fixed with 4 % paraformaldehyde in phosphate-buffered saline (PBS) for 10 min. Then, cells were permeabilized with 0.2 % Triton X-100 in PBS for 15 min, blocked 1 h with 10 % fetal bovine serum, 5 % nonfat dried milk, 0.5 % BSA and 0.1 % Triton X-100 in PBS, and incubated overnight at 4 °C in the same buffer containing anti-HA and anti-PKM2 antibodies. Samples were washed three timeswith PBS and incubated with a 1/500 dilution of anti-mouse Texas Red and anti-rabbit Alexa-Fluor 488 (Invitrogen, Madrid, Spain). Then, cellswere washed three times with PBS and mounted on slices using Aqua-Poly/Mount coverslipping medium (Polysciences, Inc. Eppelheim, Germany). Images were acquired with an uprightLeica DM RXA2microscope using an PL APO 63× oil 1.4 N.A. immersion objective, and the Leica IM50 software. At least 100 cells were counted in each condition.

### Statistical data analysis

Data are expressed as means with standard deviation. Statistical significance of differences between the groups was evaluated by a paired Student’s *t*-test with two-tailed distribution. The significance has been considered at ** *p* < 0.01, as indicated.

## Abbreviations

AMPK: AMP-activated protein kinase
PKM: Muscular isoform of pyruvate kinase
R5/PTG: Protein phosphatase 1 glycogen targeting subunit
PVDF: Polyvinylidene difluoride membranes
SC: Synthetic complete medium
SDS-PAGE: Sodium dodecylsulfate polyacrylamide gel electrophoresis
